# Education for all in the era of personalized medicine

**DOI:** 10.1186/1878-5085-5-S1-A139

**Published:** 2014-02-11

**Authors:** Meral Özgüç

**Affiliations:** 1 Professor of Medical Biology, Director, DNA/ Cell Bank for Rare Diseases, Hacettepe University Faculty of Medicine, Ankara, Turkey

## 

Genome science and related technologies is a strong contributing factor to the paradigm shift of today’s medicine. The concept of predictive, preventive and personalized medicine is beginning to find a niche in health care and the diagnosis and treatment of rare as well as common diseases will benefit from the personal genome information as the genomics technologies especially whole genome sequencing will get more affordable and more widely used. In order to increase the impact of personalized medicine, education at the level of all stakeholders is needed. One important aspect that should be taken into consideration is the very fast advances in the technologies that are used and the information dissemination that is lagging behind. Thus education and awareness raising should be done at a wide spectrum:

1- **Medical curricula** should be reviewed to meet the needs of new graduates that are confronted with new technology challanges.

2- Experts in the field of genomics need to disseminate information at the level of the **general public** to enable participation in the decision making processes.

3- **Policy makers** need to be sufficiently informed to set priorities and decide for financial allocations.

4- More **nurses, genetic counsellers** are needed who can team with medical doctors in the monitoring of patients.

**5- Ethics review committees** that review research applications involving emerging technologies have a challanging task.

Furthermore, as companies begin to offer direct to consumer tests, ethics need to catch up with the technological advances and **ethical reflections** involving the general public is needed for awareness raising. Within the concept for education for all, I would like to draw attention to the technological gap between innovative countries and developing countries where technology transfer is costly and the critical mass and infrastructures are not sufficient. Education at all levels is needed even if they lack technology platforms. Biological samples can cross borders to obtain diagnosis and interpretion and delivery of results maybe problematic. Looking towards **Horizon 2020**, it may be a good opportunity to take up the area of **education and information dissemination** as one of the holistic goals. A **global approach** to form networks between developed and less developed countries will definitely benefit all patients in the long run. EPMA is setting a good example in reaching to a wide range of stakeholders. An example is the Springer book series on different aspects of personalized medicine. A new title will be coming out in 2013; Rare Diseases: Integrative PPPM Approach as the Medicine of the Future. This book can also find high circulation in developing countries to be used as lecture material in graduate and postgraduate curricula.

